# Unraveling the Complexity of HDL Remodeling: On the Hunt to Restore HDL Quality

**DOI:** 10.3390/biomedicines9070805

**Published:** 2021-07-12

**Authors:** Leonie Schoch, Lina Badimon, Gemma Vilahur

**Affiliations:** 1 Cardiovascular Program, Institut de Recerca, Hospital Santa Creu i Sant Pau, 08025 Barcelona, Spain; LSchoch@santpau.cat (L.S.); lbadimon@santpau.cat (L.B.); 2 Faculty of Medicine, University of Barcelona (UB), 08036 Barcelona, Spain; 3 CiberCV, 08025 Barcelona, Spain; 4 Cardiovascular Research Chair, UAB, 08025 Barcelona, Spain

**Keywords:** HDL-cholesterol, HDL modifications, cardiovascular disease

## Abstract

Increasing evidence has cast doubt over the HDL-cholesterol hypothesis. The complexity of the HDL particle and its proven susceptibility to remodel has paved the way for intense molecular investigation. This state-of-the-art review discusses the molecular changes in HDL particles that help to explain the failure of large clinical trials intending to interfere with HDL metabolism, and details the chemical modifications and compositional changes in HDL-forming components, as well as miRNA cargo, that render HDL particles ineffective. Finally, the paper discusses the challenges that need to be overcome to shed a light of hope on HDL-targeted approaches.

## 1. The HDL-C Hypothesis Revisited

Epidemiological and observational studies on healthy volunteers with no baseline cardiovascular disease (CVD) [1,2,3,4] demonstrated that HDL protects from atherosclerosis and coronary artery disease [5]. Supporting this concept, multiple in vitro and experimental data demonstrated the benefits of HDL particles in the cardiovascular system. The fact that low high-density lipoprotein-cholesterol (HDL-C) was found to be associated with future coronary heart disease [6] suggested that raising HDL-C levels would provide control over the atherosclerotic process.

**Figure 1:**
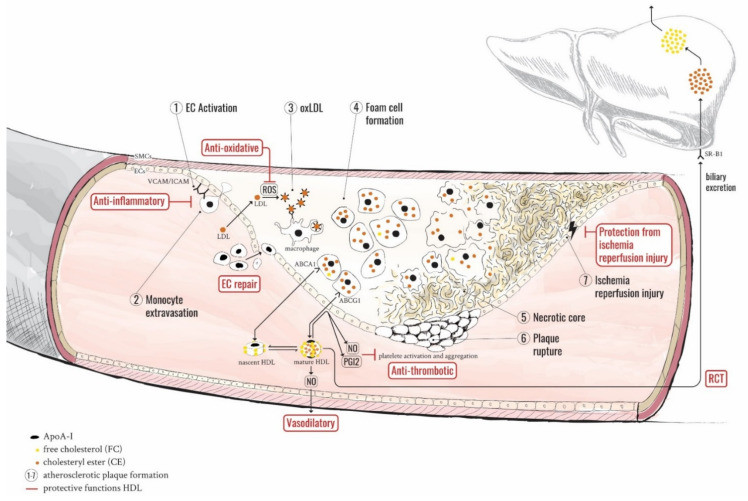
The stepwise development of atherosclerosis and the protective interference of HDLs.

Although the best-known antiatherogenic property of HDL is its ability to induce cholesterol efflux and mediate reverse cholesterol transport (RCT) [7,8,9], HDLs were also shown to exert further athero- and cardioprotective functions, which include protection from oxidative and inflammatory damage [10,11,12,13], prevention of thrombosis [14,15] and ischemia-reperfusion injury [16], as well as promotion of nitric oxide synthesis [11,17] and endothelial cell renewal [11,18] (detailed in Figure 1 and reviewed in [19]). Despite data from animal models repeatedly indicating that overexpressing human ApoA-I (as one of the key components of HDL particles) is atheroprotective in mouse [20,21] and rabbit [22,23], the involvement of other HDL components is less clear. As such, HDL-related cholesterol receptors and transporters (ATP-binding cassette transporters (ABCA1 and ABCG1) and scavenger receptor class B type 1 (SR-B1)), lipoprotein-associated enzymes (lecithin–cholesterol acyltransferase (LCAT) and lipases), and the cholesteryl ester transfer protein (CETP) have proven harder to directly link to benefits in animal models [5]. Special care should be taken with differences in species regarding certain aspects of lipid metabolism as, for example, rodents and pigs express mRNA for CETP but do not secrete it into the plasma.

Mendelian randomization studies have not confirmed an association between increased CV risk and HDL-C levels [24], and drugs aimed at raising HDL-C for secondary prevention (niacin, and CETP inhibitors) have not reduced CV events [25,26,27,28,29]. Furthermore, Madsen et al. described a study including a combined 116,508 individuals from the Copenhagen General Population Study and the Copenhagen City Heart Study which found that extremely high HDL-C levels are paradoxically associated with high all-cause mortality. As such, the association between HDL-C and all-cause mortality follows a U-shaped curve with both extremely high and low HDL-C concentrations being associated with increased mortality in both men and women [30]. Potential explanations behind these findings have been postulated: extremely high concentrations are often due to genetic variants [31] which are associated with a high risk of coronary heart disease (e.g., mutations in *LIPC* and *SCARB1*) [32], and the functionality of HDL in individuals with extremely high HDL-C may be compromised and even cause harm. As such, over recent years doubts have arisen concerning the causative involvement of HDL-C in CV protection. Moreover, discussion has intensified regarding whether HDL-C is an adequate measure for HDL-conferred protection or whether HDL particle numbers or HDL function would be better representatives [19,33].

The numbered steps indicate the progressive development of the atherosclerotic plaque and the protective functions of HDL particles are highlighted in red. Atherosclerotic development starts with stress-induced activation of vascular endothelial cells (ECs), which results in elevated levels of adhesion molecules such as intracellular cell adhesion molecule (ICAM) and vascular cell adhesion molecule (VCAM). Circulating monocytes are attracted and by interacting with these endothelial receptors, they extravasate to the site of inflammation within the intima (steps 1 and 2). HDL particles counteract this initiating step by repressing the expression of endothelial adhesion molecules, subsequently preventing monocyte infiltration. In addition, HDLs help to prevent apoptosis and contribute to EC repair by recruiting endothelial progenitor cells. Otherwise, activated monocytes differentiate into tissue-resident macrophages and produce cytokines, as well as reactive oxygen species (ROS), which convert low-density lipoproteins (LDLs) into their most pro-atherogenic form, oxidized LDLs (oxLDLs) (step 3). HDL particles carry potent antioxidative enzymes such as paraoxonase 1 (PON1) and PAF-acetylhydrolase which protect both HDL- and LDL-associated components against oxidation. In the progression of disease, increasing lipid deposition and/or decreasing cholesterol efflux overwhelm macrophages in their task to clear the accumulated oxLDLs and turn them into foam cells (step 4). One of the best-studied properties of HDLs is their capacity to efflux cholesterol from those lesion site macrophages/foam cells and transport it to the liver for biliary excretion, a process known as reverse cholesterol transport (RCT). Without alleviation, these foam cells eventually undergo apoptosis and generate the characteristic necrotic core of progressing plaques (step 5). If the fibrous cap separating the instable plaque from the bloodstream ruptures, the content of the necrotic core immediately provokes platelets to clot and form a thrombus (step 6). Starting with a minimal impact on the vessel diameter by a fatty streak, plaque rupture with subsequent thrombosis leads to far more severe obstructions of the blood flow. To prevent the circulatory system from experiencing a major event, HDLs induce ECs to produce nitric oxide (NO) and prostacyclin (PGI2), which not only result in inhibited platelet activation and aggregation but also vasodilation which counterbalances the constricted vessel diameter. Complete obstruction of the vessel leads to ischemia-reperfusion injury, a condition which in turn leads to oxygen deprivation behind the thrombus, and acidosis and accumulation of metabolic waste products before the blockage. When the obstruction is resolved and blood flow restored, the newly available oxygen leads to additional oxidative damage to the surrounding cells before it can provide its life-saving properties. SMC: smooth muscle cell; EC: endothelial cell; VCAM: vascular cell adhesion molecule; ICAM: intracellular cell adhesion molecule; LDL: low-density lipoprotein; oxLDL: oxidized LDL; ROS: reactive oxygen species; NO: nitric oxide; PGI2: prostacyclin; HDL: high-density lipoprotein; ABCA1/ABCG1: ATP-binding cassette transporters A1/G1.

**Figure 2:**
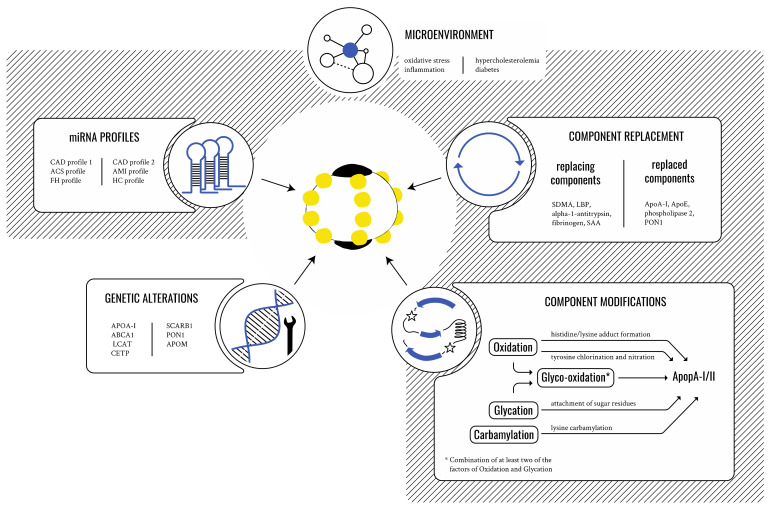
Characteristics and influencing factors of dysfunctional HDL particles.

Emerging knowledge associates several comorbidities such as hypercholesterolemia, diabetes mellitus, renal dysfunction, and oxidative and inflammatory stress with dysfunctional HDL particles and altered HDL composition. Consequently, the “HDL quality over quantity hypothesis” gained popularity as a possible explanation for missing protective effects in clinical trials [33,34,35]. The scientific community has accordingly redirected its efforts towards deciphering the HDL particle’s structure, function, and its remodeling capacity. The implementation of “omic” technologies has allowed for the identification of the modifications, changes, and replacements that HDL particles undergo under pathological conditions that negatively alter HDL function (Figure 2).

It is critical that we gain in-depth understanding of HDL particle component modifications, molecular cargo (including miRNAs), and remodeling factors which render HDL particles dysfunctional in pathological conditions. Knowledge of the mechanisms involved in HDL loss of function will be instrumental to restoring the CV protection afforded by HDL particles.

The microenvironment plays a critical role in the generation of dysfunctional HDL particles by promoting alterations in HDL composition. Dysfunctional HDLs exert distinct miRNA profiles and have key components replaced and/or modified. HDL protective functions can also be impaired by rare genetic alterations. CAD: coronary artery disease; ACS: acute coronary disease; FH: familial hypercholesterolemia; AMI: acute myocardial infarction; HC: hypercholesterolemia (animal model); APOA-I: apolipoprotein A-I; ABCA1: ATP-binding cassette A1; LCAT: lecithin cholesteryl acetyltransferase; CETP: cholesteryl ester transfer protein; SCARB1: scavenger receptor class B member 1; PON1: paraoxonase 1; APOM: apolipoprotein M; SDMA: symmetric dimethylarginine; LBP: lipopolysaccharide-binding protein; SAA: serum amyloid A; ApoE: apolipoprotein E.

## 2. HDL Particle Alterations: On the Lookout for What Makes HDL Particles Lose Their Protective Functions

Lifestyle modifications such as increased physical activity, weight loss, dietary changes [36], smoking cessation, etc. have been shown to increase HDL-C by 20–30% [37] and exert beneficial effects on HDL protective functions such as increased cholesterol efflux and antioxidant and anti-inflammatory capacity, as well as elevated eNOS expression/NO production and vasodilation [38,39,40,41]. However, most of the conducted studies measured HDL-C/LDL-C/triglyceride levels rather than HDL composition and protective functions. The fact remains that it is difficult to ensure adherence to exercise and dietary programs, leading to variable results and difficult data interpretation. It is also challenging to separate the effects of dietary interventions from those of weight loss, as both tend to go hand in hand [42,43,44]. In fact, active weight loss was reported to decrease HDL-C levels, while stabilized lower weight seems to increase HDL-C [42]. A direct association has also been established between HDL and the protective adipokine adiponectin, which was shown to increase following dieting or exercise [45].

Negative effects, on the other hand, were shown to be caused by the presence of metabolic and inflammatory diseases which affect HDL components and cargo with potential consequences on CV protection.

### 2.1. HDL Component Modifications

The HDL particle composition is highly complex. Its functional properties strongly depend on the composition of proteins (>85 identified) [46], lipids (>200 identified species) [47], hormones, vitamins, and miRNAs [48], all contributing to its biological functions. Over recent years, significant efforts have been made to untangle HDL’s composition, its susceptibility to changes, and the consequent impairment of protective functions.

#### 2.1.1. Oxidation

One of the best-known modifications is oxidation, a process that can be promoted by a variety of stimuli and primarily affects lipid and protein entities. Comparisons of oxidation kinetics have shown that HDL and LDL profiles are very much alike, suggesting similar mechanisms and oxidation rates [49]. HDL-associated lipids are just as prone to being oxidized as their LDL counterparts. HDLs, however, carry antioxidant enzymes such as paraoxonase (PON)1 and lecithin-cholesterol acyltransferase (LCAT) which can counteract oxidation [10]. The most frequently found oxidized phospholipid in HDLs is phosphatidylcholine, which can be catalytically transformed into lysophosphatidylcholine, a metabolite that not only can be transferred to LDL particles, where it promotes atherogenesis, but can also impair SR-B1-mediated RCT when accumulating in HDLs [50]. Reactive metabolites from phospholipid oxidation have also been shown to selectively modify ApoA -I and -II, the most important structural protein components of HDLs. While Gao et al. reported a covalent histidine-linked Michael adduct at H155, H162, H193, and H199 in helices 6–8 of ApoA-I to be the most abundant modification by endogenous oxidized phospholipids [51], Martínez-López et al. identified Trp50 and Trp108 as the most significantly oxidized residues with significant cholesterol efflux capacity (CEC) impairment in patients with abdominal aortic aneurysm [52]. Another mechanism by which ApoA-I may suffer direct oxidation relies on myeloperoxidase (MPO), an enzyme overexpressed in atherosclerotic lesions [53]. The MPO-oxidized ApoA-I methionine residues (Met(O)-ApoA-I) were recently identified to lead to a paradox. While Met(O)-ApoA-I monomers increase CEC, they also initiate amyloidogenesis which ultimately results in the sequestration and inactivation of otherwise antiatherogenic and HDL-forming ApoA-I [54]. Met86(O)-ApoA-I and Met148(O)-ApoA-I have been reported to maintain their cholesterol-accepting capacity while strongly inducing pro-inflammatory cytokine production in surrounding immune cells [55], contributing to atherosclerotic disease progression. MPO overexpression in atherosclerotic lesions is also known to result in chlorination and nitration of ApoA-I tyrosine residues. Especially, chlorination of Tyr192 has been shown to impair ABCA1-dependent RCT [56,57,58] and markedly increase atherosclerotic plaque instability [59]. ABCA1-dependent RCT was also shown to be impaired by MPO-mediated oxidation of the Trp72 residue (Trp72(O)-ApoA-I) [60], which, interestingly, accounted for 20% of the ApoA-I in atherosclerotic arteries, while the overall abundance in circulation was low [61]. Elevated Trp72(O)-ApoA-I levels demonstrated a potent pro-inflammatory activity on endothelial cells and were associated with increased CV risk [61].

#### 2.1.2. Carbamylation

Carbamylation is another major protein modification caused by an irreversible interaction between isocyanic acid and amino groups of proteins. Isocyanic acid is produced during urea catabolism and inflammation-induced MPO activity, which can be found at atherosclerotic lesion sites [53]. Interestingly, Holzer et al. discovered that, while MPO activity is responsible for oxidation and carbamylation of ApoA-I, the carbamyl lysine content was 20-fold higher than chlorotyrosine levels in HDLs isolated from atherosclerotic lesion sites [62]. One carbamyl lysine residue per ApoA-I was shown to be sufficient to promote SR-B1-dependent cholesterol accumulation and lipid droplet formation in macrophages [62], as well as suppress LCAT and PON1 activity, impairing HDL protective functions [63]. The carbamyl lysine content correlates with atherosclerotic lesion severity [62] and independently predicts cardiovascular risk [64].

#### 2.1.3. Glycation

Another common protein modification is glycation. HDL glycation is typically found in patients with diabetic backgrounds and is characterized by covalent attachment of sugar residues. Glycated HDL particles induce endothelial cell apoptosis and increase oxidative stress [65,66] and smooth muscle cell proliferation and migration [67]. Glycation of ApoA-I seems to lead to conformational changes at the site for LCAT activation [66,68] and reduces HDL CEC [69] and its ability to inhibit the expression of adhesion molecules [70]. In patients with type 2 diabetes, glycated ApoA-I is associated with the severity of CAD and coronary artery plaque progression [71,72] and significantly reduces ApoA-I half-life [73]. Additionally, studies in diabetic patients demonstrated that HDL can be glyco-oxidized, a state that combines glycating and oxidizing modifications. Glyco-oxidized HDLs were shown to carry significantly reduced amounts of polyunsaturated fatty acid species, while palmitic, stearic, and oleic acid levels were kept elevated. Modified linoleic acid-containing phospholipids such as PC have been suggested to be responsible for the significantly increased ability to inhibit collagen-induced platelet activation and aggregation by SR-B1 [74]. A possible explanation for some controversial results is the potentially different impact of varying degrees of oxidation and glycation on HDL function. The biophysical properties of phospholipids, sphingolipids, free cholesterol, apoproteins, and triglycerides are key to sustaining the homeostasis of membrane fluidity. Given its modifications, or even loss of key components, membrane fluidity is especially vulnerable to changes. Subsequently, it affects the ability to induce cholesterol efflux, supposedly by impairing efficient interaction with receptors or transporters involved in cholesterol exchange [75,76].

In summary, the modification of endogenous HDL components primarily results in the loss of HDL-protective functions and is caused by adverse environmental conditions such as oxidative stress and high glucose levels, always taking into consideration that oxidation of the protein species seems secondary to reactive metabolite formation by lipid oxidation.

### 2.2. HDL Component Replacements

Replacement of physiological-state HDL constituents by acute-phase proteins is a process strongly associated with chronic inflammation, an underlying condition in many diseases including atherosclerosis and metabolic disorders. Within the acute-phase proteins found in modified HDL, such as lipopolysaccharide-binding protein, alpha-1-antitrypsin, and fibrinogen, serum amyloid A (SAA) is probably the best-studied [77]. The incorporation of acute-phase proteins involves the reciprocal replacement of ApoA-I, a reduced activity of antioxidant enzymes such as PON1 and LCAT, and the accumulation of inflammatory enzymes and reactive metabolites of lipid oxidation such as the aforementioned MPO [77]. The precise pathophysiological role of SAA remains unclear, but the functional changes in SAA-containing HDLs may shed some light. SAA-containing HDL particles seem to exert reduced RCT, decreased hepatic cholesteryl ester uptake due to competitive binding of lipid-free SAA to SR-B1, and blockade of the hepatic binding site for interaction with HDLs [78]. Schuchardt et al. recently demonstrated that HDL-bound SAA does not only reduce HDL anti-inflammatory properties, but actually activates pro-inflammatory toll-like receptors (TLR2/4), promoting vascular inflammation [79]. Additionally, SAA-HDLs are trapped at sites of vascular lesions due to increased interactions with extracellular matrix proteoglycans [80], an interaction that increases their risk of oxidation [81]. The combination of reduced ApoA-I levels and oxidized HDL components (ApoA-I and phospholipids) may account for the observed reduction in CEC and anti-inflammatory properties of HDLs carrying SAA.

PON1 is another important HDL-associated factor that is diminished during an acute-phase response. While there is evidence for decreased antioxidant activity of PON1, the exact mechanism by which it occurs remains elusive. Suggestions range from replacement by acute-phase proteins [82,83] and suppressed expression [84] to a mere shift from PON to arylesterase enzymatic activity [85]. In line with the replacement approach, both PON1 and SAA have been shown to preferentially co-isolate with dense HDL3 particles [86,87]. Therefore, PON1 on the protective HDL3 particle might be replaced by SAA in the acute-phase HDL3 particle.

Interestingly, patients with below-median levels of SAA and symmetric dimethylarginine (SDMA) presented with the traditional inverse correlation between HDL-C and CVD mortality. In contrast, those with above-median levels were reported to show the contrary [88,89]. In line with these results, other inflammatory markers such as hsCRP (acute-phase protein) and ApoC-III (a marker for unfavorable outcome) have been confirmed as independent risk predictors for CVD [90,91]. Consequently, patient stratification based on their inflammatory burden may provide an easy measurement for HDL dysfunction with a positive correlation between acute-phase proteins/inflammatory markers and CVD outcomes. However, if SAA ought to function as a potential therapeutic target beyond a mere biomarker, the causative involvement of SAA needs to be addressed.

Besides chronic inflammation, we identified hypercholesterolemia to affect HDL composition and adversely impair HDL functionality. HDLs formed under high LDL-cholesterol levels undergo structural remodeling which, in turn, is associated with the impairment of HDL-mediated cardiovascular protective functions [92]. In particular, we revealed by “omic” approaches that HDLs formed under high LDL-cholesterol levels showed altered lipid and protein profiles as compared with native HDL particles with depleted phosphatidylcholine species at the surface, enriched cholesteryl ester in the core, and reduced contents of key cardioprotective proteins (retinol binding protein 4, ApoM, and the cellular retinoic acid binding protein 1) [93]. Most interestingly, these changes were associated with a loss in HDL antioxidant potential and CEC, as well as impaired protection against ischemia-reperfusion injury in a preclinical model of myocardial infarction [94]. In line with these results, HDL particles from patients with coronary heart disease or type 2 diabetes mellitus were reported to carry significantly reduced levels of the phospholipid species phosphatidylinositol (36:2, 34:2) and phosphatidylcholine (36:2, 34:2) [95], both major components of the HDL lipidome.

Furthermore, it was proven that the infusion of native and functional HDL particles into hypercholesterolemic animals does not confer cardioprotection, suggesting a rapid dyslipidemia-mediated adverse remodeling of HDL particles [96]. Hafiane et al. confirmed that HDL remodeling occurs very quickly, and that HDL-mediated cholesterol efflux (as a readout of HDL remodeling) remains impaired even three months after ACS [97]. These findings suggest that the recovery from acute-phase response changes may take much longer than anticipated. As such, the underlying problem does not seem to be a single factor but sustained exposure to athero-prone stimuli (e.g., hypercholesterolemia, inflammation, oxidative stress, or disturbed glycose levels) that induce a multitude of changes that render HDLs dysfunctional or even pro-atherogenic. In this regard, besides losing their cardioprotective potential, we recently demonstrated in a preclinical rabbit model of atherosclerosis that hypercholesterolemic HDLs also lose their ability to regress and stabilize atherosclerotic plaques and may even increase plaque burden [98].

Finally, HDL particles isolated from patients with established CAD proved that, instead of replacement, redistribution of important components could also affect HDL’s protective function. HDL particles containing ApoE were shown to robustly associate with reduced CV risk [99]. However, CAD patients demonstrated a redistribution of ApoE from its physiological site of residence on HDL2 particles to the HDL3 counterpart, with the suggested effect of impairing cholesterol efflux [100]. HDL particles can be subdivided by density into two main subfractions: large, light, lipid-rich HDL2 and small, dense, protein-rich HDL3 particles [101]. The HDL3 subfraction is the generally more potent, with higher anti-thrombotic, antioxidant, anti-inflammatory, and anti-apoptotic capacities, as well as ABCA1-mediated cholesterol efflux [101,102]. In line with the aforementioned data, low HDL3-C levels are associated with a >50% higher risk for major cardiovascular events in secondary prevention, while HDL2-C and HDL-C are not [103]. Finally, the enzymatically truncated ApoA-IΔ(1-38) isoform has been linked to a significantly lower antioxidant capacity and preferential binding to LDL instead of HDL particles in diabetes patients with increased cardiovascular risk [104].

### 2.3. HDL miRNA Profile

HDL particles carry miRNAs, yet their functional implications on HDL are not fully known. Table 1 summarizes HDL-miRNA profiles that have been associated with CVD. Interestingly, the studies cited in Table 1 show contradictory data as to whether a specific miRNA is up- or down-regulated, as seen for miR-92a and miR-146a. A possible explanation for the discrepancy between the profiles in CAD and ACS could relate to their clinical background. HDL-miRNA profiles may subsequently reflect the effect of an acute event or a chronic compensatory mechanism that develops over the course of disease onset and progression. However, there are methodological factors, e.g., methods of isolating HDLs, isolating RNA, and analyzing miRNA, that could affect the results.

We reported in a preclinical animal model that HDL particles formed in the presence of diet-induced hypercholesterolemia carry a high content of miR-126, which reduces the expression of proteins that modulate cell survival upon delivery to endothelial cells through an SR-B1-related mechanism [105]. In this regard, we and others have identified an SR-B1-dependent mechanism of delivery for HDL-miRNAs and confirmed that physiological levels of transported miRNAs are sufficient to affect downstream target regulation [105,106]. In contrast, however, Wagner et al. questioned the functional relevance of HDL-transferred miRNAs due to very low transfer rates [107].

### 2.4. Genetic Alterations Affecting HDL

Besides the influence of the patient’s comorbidities on HDL remodeling, genetic alterations in HDL metabolism-related components were also shown to affect HDL potential (Figure 2). Nevertheless, it is important to emphasize that the overall impact of HDL-related genetic defects on CVD is much lower than lifestyle- and comorbidity-associated factors. Table 2 summarizes those genetic alterations that result in impaired HDL function and may impact CVD.

## 3. Conclusions

The complexity of the HDL particle and its vulnerability to modification offer countless opportunities for the HDL particle to be used in therapy. Increasing our knowledge about HDL component modifications (e.g., oxidation, glycation, and carbamylation of ApoA-I) and compositional changes (e.g., the incorporation of acute-phase proteins instead of antioxidant enzymes or ApoA-I molecules, and changes in miRNA profile) is essential as it holds great potential to identify why, how, and with what consequences HDL particles are rendered dysfunctional. Once the causes are found and the triggers identified, HDL dysfunctionality will be much better defined, allowing us to restore HDL functionality and providing a future for HDLs in CV therapy. HDL-based therapeutic approaches were applied to resolve the residual risk following statin treatment by raising HDL-C levels. They were unsuccessful due to a lack of understanding of the HDL particle and the reason why a particle carrying large amounts of cholesterol (indeed this is what HDL-C means) was of no benefit. Research to identify the essence of HDL particle quality will be instrumental in uncovering new targets and applying innovative new therapies to CVD.

## Figures and Tables

**Table 1:** CVD-associated miRNA profiles of HDLs.

Disease	miRNA Profile		Function	Ref
	*upregulated*	*downregulated*		
CAD	miR-33a miR-92a * miR-125a miR-146a † miR-486		miR-33a: inhibits genes involved in cholesterol transport and fatty acid metabolism (decreased cholesterol efflux and fatty acid oxidation). Additionally promotes cardiac fibrosis by targeting matrix metalloproteinase 16. miR-92a: induces endothelial dysfunction and cardiomyocyte apoptosis. miR-125a: inhibits vascular smooth muscle cell proliferation and migration by targeting MAPK1. Endothelial cell metabolic reprogramming (glycolysis) mediates miR-125a-induced vascular hyperbranching. miR-146a: associated with the control of inflammatory processes. miR-486: increases cholesterol accumulation in foam cells. Hypoxia-induced expression. Additionally inhibits cardiomyocyte apoptosis.	[108]
ACS		miR-30c miR-92a * miR-146a †	miR-30c: downregulates the pro-fibrotic connective tissue growth factor, modulating structural changes in the extracellular matrix of the myocardium. miR-92a: pro-inflammatory and angiogenesis-promoting. Highly expressed in endothelial cells. miR-146: see above.	[107]
FH	miR-105 miR-106a miR-223		miR-105: disrupts vascular integrity (Zonula Ocludens-1 tight junctions). miR-106a: induces cardiac hypertrophy. miR-223: potentially atherogenic and predictive for coronary artery disease. Direct targets: Ras homolog family member B (controls endothelial barrier integrity during inflammation) and ephrin A1 (pro-angiogenic upon hypoxia).	[106]
FH	miR-486 miR-92a *		miR-486: see above. miR-92a: see above.	[109]

CAD: coronary artery disease; HDL: high-density lipoprotein; ACS: acute coronary syndrome; FH: familial hypercholesterolemia; MAPK1: mitogen-activated protein kinase 1; */†: miRNAs found in more than one profile.

**Table 2:** Genetic alterations in HDL metabolism-associated factors.

Gene	Annotated Mutations	Associated to CV Risk?	Affected Physiological Parameters	Ref
APOA-I	83	Yes	Mild (heterozygous) to almost complete absence (homozygous or compound heterozygous) of ApoA-I and HDL-C with a predisposition for premature CVD. (Hereditary) amyloidosis due to the accumulation of abnormal N-terminal ApoA-I fragments.	[110,111,112,113,114]
ABCA1	268	Yes	Heterozygous loss-of-function: common in people with low HDL-C presenting with a 50% reduction in cholesterol efflux and moderately reduced HDL-C. Homozygous: Tangier Disease counts 100 cases worldwide and shows drastic impairment of cholesterol efflux and hardly any plasma HDL-C and ApoA-I. ABCA1 mutations seem to be dominant: combinations with mutations that increase HDL-C levels, sustain very low HDL-C. Often accompanied by neurologic, ophthalmologic, dermatologic, hematologic, and histiocytic symptoms.	[115,116,117]
LCAT	117	Expected, but not confirmed due to low case numbers and high heterogeneity.	Mild (fish-eye disease) to severe (familial LCAT deficiency) loss of enzymatic activity resulting in a reduction of ApoA-I and HDL-C plasma levels of up to 80%.	[118,119,120]
CETP	71	Yes, but extent strongly depends on the respective mutation.	Partial to complete deficiency increases ApoA-I and HDL-C plasma levels. More frequently found in the Japanese population.	[30,121,122,123,124]
SCARB1	18	Unclear. Yes for rs4238001 and p.P376L (almost exclusive to Ashkenazi Jews).	Increased HDL-C (impaired hepatic uptake) and foam cell formation (impaired cholesterol efflux). A higher prevalence in the Icelandic population.	[105,125,126,127]
PON1	22	Unclear. Yes for Q192R and V109I (ischemic events, CAD) and suggested for L55M (AS) in diabetic patients.	Protection from oxidation diminished by impaired enzymatic activity (Q192R) or reduced concentrations (L55M).	[128,129,130,131,132,133,134]
APOM	5	Yes for T778C, T1628G, T855C, C724del.	Supposedly down-regulated ApoM expression and elevated total cholesterol levels. Almost exclusively identified in the Han Chinese population.	[135,136,137]

APOA-I: apolipoprotein A-I; HDL-C: high-density lipoprotein cholesterol; CVD: cardiovascular disease; ABCA1: ATP-binding cassette transporter A1; LCAT: lecithin–cholesterol acyltransferase; CETP: cholesteryl ester transfer protein; SCARB1: scavenger receptor class B member 1; PON1: paraoxonase 1; APOM: apolipoprotein M.
